# Experiences providing care to patients with mental health conditions in a tertiary care hospital emergency department in Jerusalem

**DOI:** 10.1371/journal.pone.0340973

**Published:** 2026-01-16

**Authors:** Zvika Orr, Levi Jackson, Evan Avraham Alpert, Mark D. Fleming

**Affiliations:** 1 Selma Jelinek School of Nursing, Jerusalem College of Technology, Jerusalem, Israel; 2 Department of Emergency Medicine, Hadassah Medical Center- Ein Kerem, Jerusalem, Israel; 3 Faculty of Medicine, The Hebrew University of Jerusalem, Jerusalem, Israel; 4 School of Public Health, University of California Berkeley, Berkeley, California, United States of America; Bar-Ilan University, ISRAEL

## Abstract

The emergency department (ED) often serves as the first point of care for those with mental health conditions. Mental health-related visits to the ED tend to increase during and after public health crises. In Israel, the war that started in 2023 has had substantial adverse effects on the population’s mental health, increasing the need for emergency services for people with mental health conditions. This article examines the perceptions and experiences of Israeli staff providing care to patients with mental health conditions in an ED of a tertiary-care hospital in Jerusalem. Based on an inductive thematic analysis of 24 semi-structured interviews with staff members, this study sheds new light on the staff’s challenges in treating these patients. The study found that providers navigated a high level of stigma towards people with mental illness. Many providers were aware that negative perceptions of these patients were potentially harmful and may lead to diagnostic overshadowing, and in some cases, they tried to mitigate the effects of stigma. Staff often viewed patients with mental illness as inappropriate users of the ED, assuming limited responsibility for these patients. The findings also illuminate the providers’ inadequate training and skills for treating and managing mental health, as well as organizational and structural constraints. The article recommends ways to improve the treatment of mental health in EDs, such as educational workshops, more support of mental health specialists in EDs, providing calm environments, working alongside experts by experience, and conducting person-centered risk assessments. EDs should strengthen collaboration and referral pathways to community-based mental health services. Moreover, the healthcare system must provide patients with alternative sources of care, such as community crisis centers. These steps can mitigate the expected post-war mental health crisis in Israeli EDs and are relevant to many other countries.

## Introduction

In recent years, there has been a global rapid growth in mental health problems. It is estimated that in the United States, one in five adults (52.9 million) has a mental health disorder [1]. Mental health-related emergency department (ED) visits have increased dramatically and now account for 12.3% of all ED visits made by adults in the United States [1,2]. This increase spans all categories of mental health diagnoses, including mood disorders, anxiety disorders, psychosis and schizophrenia, suicide attempts or ideation, substance use disorders, and others [2,3]. In Israel, from 2015 to 2022 there was a 33% increase in the number of mental health-related ED visits [4]. Despite this rapid increase, EDs are often not professionally equipped to provide optimal and effective care to patients with mental health conditions [5–9]. More research is needed to understand and address this global problem.

This problem is even more acute during public health crises, when ED visits of people with mental health conditions tend to increase. For example, in the United States, ED visit rates for mental health conditions, suicide attempts, and drug and opioid overdoses increased during the COVID-19 pandemic [10,11]. In many countries, this trend continued even post-pandemic [12]. In Israel, the war that started in October 2023, which has led to a severe public health and humanitarian crisis in Gaza, has had substantial adverse effects on the Israeli population’s mental health [13–18]. For example, during the war, the ten directors of Israel’s psychiatric hospitals stated that the country’s mental health system is about to collapse. They estimated that following the war, 300,000 additional Israeli citizens as well as soldiers returning from the battlefield will need professional mental health care [19]. Patients face waiting times of over six months for an initial psychiatric treatment [18], and many are unable to obtain an appointment at all [20]. Against this backdrop, more patients with mental health problems are presenting to the ED to seek prompt psychiatric care.

While the ED has become an increasingly important site for receiving mental health care, in many countries, most patients who receive mental health ED services report negative experiences [5,7,21,22]. They feel that the staff trivialize their distress, show low empathy, see them as a burden, disrespect them, do not listen to them, and do not take them seriously [5,21,23]. Patients experience judgment, stigma, and discrimination when presenting to the ED, resulting in feelings of powerlessness and hopelessness [6,23]. Some patients report perceiving punitive attitudes from staff in response to self-harming or suicidal behaviors, including being blamed for committing a sin, seeking attention, or wasting the provider’s time [21].

The poor experiences of patients seeking mental health care in the ED are caused by multiple factors, including negative and stigmatizing views among ED staff towards patients with mental health conditions [6,9,12,21] and lack of training and confidence in treating mental health conditions and managing patients who express aggressive behaviors [24,25]. For instance, ED nurses reported a lack of educational preparation for treating patients with ill mental health, as well as a lack of continuing education in this field [22,24,26]. The ED environment is often inappropriate for people with mental health issues, as this is a high-pressured, over-stimulating, noisy, uncomfortable, fast-paced environment, lacks privacy, and is characterized by long waiting times [21,27]. Further, organizational limitations contribute to poor care, including the absence of specific mental health protocols and triage tools, lack of effective interventions, inadequate time required for giving full attention to patients with mental health conditions, workload and dealing with competing priorities, insufficient specialized psychiatric staff, and limited referral pathways [12,21,22,24,26,28].

This emerging body of research notwithstanding, several key aspects of this subject have remained under-researched. Scholars pointed to the need for more studies that will comprehensively investigate ED staff experiences caring for patients with mental health conditions, including their suggestions on future professional practices and ways of organizing future care in the ED [29]. Moreover, very little is known about this subject in Israel. It is important to examine the provisioning of mental health care in the ED in Israel because of the growing volume of patients exacerbated by the lack of resources in the community and the growing need related to the ongoing war.

This article aims to contribute to bridging these global and local knowledge gaps and thus help ameliorate said poor outcomes of ED care for patients with mental health presentations. The article examines the perceptions and experiences of Israeli ED staff providing care to patients with mental health conditions in a tertiary-care hospital in Jerusalem. Data were collected before the outbreak of the war. This is the first study to explore these issues in Israel, and we propose that its findings are relevant to many other countries.

### Mental health in Israel

Israel provides universal healthcare coverage for all citizens, based on the National Health Insurance Law [30]. In 2015, Israel implemented a mental health reform that transferred the responsibility for providing mental health services from the Ministry of Health to the four public health maintenance organizations (HMOs). However, many issues remained unresolved after the reform [31–34]. The 130,000–150,000 people with severe mental illness in Israel [32] show higher hospitalization rates compared to the total population, and this decades-long disparity increased in the years following the reform (2015–2019) [35]. Only 30,000 persons receive community psychiatric rehabilitation services, constituting around one-fifth of the eligible population [36]. Furthermore, mental health in Israel receives only 5.2% of the national health budget, compared to 10%–16% in high-GDP Western countries [34]. In contrast to general medicine – where 63% of spending is publicly funded and 37% privately funded – the distribution in mental health is approximately the reverse [34].

Moreover, there is a significant shortage of mental health professionals in Israel, particularly within the public healthcare system. For example, in 2022, there were 1,237 licensed psychiatrists under the age of 67 in Israel – a modest increase of approximately 9% since 2010, compared to a 25% increase in the general population over the same period [37]. The ratio of practicing psychiatrists in Israel is 9.8 per 100,000 people – nearly half the average rate in developed countries, which stands at 18.9 per 100,000 [37]. According to the chair of the Forum of Directors of Mental Health Centers, as of December 2023, the Israeli mental health system faced a shortage of approximately 400 psychiatrists. This shortfall is expected to double by the end of 2028, as a substantial portion of the workforce nears or has already reached retirement age [38].

All these factors likely increase and will continue to increase the use of ED services by patients with mental illness. Emergency psychiatric services are provided both in the EDs of general hospitals and psychiatric hospitals [39]. Both settings are impacted by the broader systemic challenges.

The protracted Israeli-Palestinian armed conflict has major adverse effects on the mental health of Palestinians and Israelis [40–44]. Specifically, the current Israel-Hamas war has an adverse impact on the mental health of both Palestinians and Israelis [13–18,45–50]. Among Israelis, the war results in a high prevalence of mental health problems, such as PTSD, anxiety, and depression, in both adults and children [13,14,16,18]. In a survey administered during the war, parents said that 83% of children exhibited high levels of emotional distress, such as anxiety (62%). Forty-eight percent of the parents reported that they themselves are struggling with anxiety and depression [13]. Another survey, which was administered twice – before and during the war – found that probable PTSD prevalence almost doubled from 16.2% to 29.8%, the prevalence of probable generalized anxiety disorder increased from 24.9% to 42.7%, and the prevalence of probable depression increased from 31.3% to 44.8% [14]. Furthermore, this survey found that minority groups are more affected by the war. Compared with Jewish citizens, Arab citizens of Israel presented with a significantly higher prevalence of probable PTSD, depression, and anxiety during the war, controlling for other variables [15,17]. Individuals experiencing economic hardships, as well as women, demonstrated higher rates of anxiety, depression, and PTSD symptoms during the war [17,18]. The Israeli Ministry of Health projected that, following the war, the number of individuals in need of mental health care would double – from approximately 340,000–680,000 [18].

As mentioned earlier, it has become more difficult to get an appointment with a psychiatrist or psychologist. According to the HMOs’ data, the average waiting time for diagnosis and initiation of mental health treatment is approximately six and a half months [18]. Physicians report that in many cases, when they refer patients to a psychiatrist or psychologist, patients are unable to get an appointment at all [20]. Consequently, an increasing number of patients with mental illness are turning to the ED for timely mental health care. We anticipate that this trend will likely increase after the war, based on the Ministry of Health’s estimates of post-war morbidity and unmet patient needs [18].

This alarming situation is well-documented in the United States, where only half of the adults with a mental health disorder receive services, and consequently, many receive their care in EDs [1,2]. Patients present to the ED with all categories of mental health disorders, the most prevalent being alcohol-related diagnoses, mood disorders, and anxiety disorders, while substantial numbers also involve psychosis and schizophrenia, suicide attempts or ideation, and other psychiatric and substance-related conditions [2]. In Israel, these ED visits only add to a pre-war situation in which many ED visits for mental health concerns are ineffective. A study in a large hospital in Jerusalem found that 57.4% of the ED visits that included a psychiatric examination were deemed “potentially ineffective,” possibly being more suitable for a psychiatry facility [39]. Furthermore, due to the war and the growing mental health prevalence, a higher proportion of people who present with physical illness in the ED also experience mental health conditions. Taken together, these recent developments require a deeper understanding of Israeli ED staff’s perceptions of and interactions with patients with mental health problems.

## Materials and methods

### Design

This qualitative study relied on the interpretivist research paradigm, and applied the interpretative phenomenological analysis approach to guide the data collection [51,52]. In keeping with this methodological approach, this article is based on 24 semi-structured, individual interviews with medical staff members in the ED of a tertiary-care hospital in Jerusalem.

This study was conducted in accordance with the Declaration of Helsinki and received ethics approval from the Institutional Review Boards of University of California, Berkeley (reference number 2018-06-11143) and the examined hospital (reference number 154−18). All research participants gave written and oral informed consent to participate in this study prior to their inclusion in the study. Please see Supplement 1 for Standards for Reporting Qualitative Research.

### Data collection

The interviewees included 9 physicians, 9 nurses, 2 physician assistants, 3 nursing students employed by the hospital in care support roles, and one employee who requested to withhold information regarding her position in the department. We approached all the department’s physicians, nurses, physician assistants, and student employees (approximately 130 staff members) and 24 (18.5%) agreed to participate in the research. Refusal to be interviewed was usually explained by a lack of time or unwillingness to discuss this study’s issues. Thirteen participants (54%) were female and eleven (46%) were male. Twenty-three participants (96%) were Jewish and one (4%) was Arab. This figure is similar to the percentage of Jews and Arabs among the physicians, nurses, physician assistants, and student employees in this ED. The participants had a wide range of professional experience, ranging from one year to four decades.

The participants were asked about their personal ED experiences and perceptions. Some questions were general, not asking specifically about patients with mental health conditions. For example, participants were asked about “a particularly challenging patient” and “the main challenge in their work.” In response to these general questions, many participants chose to explicitly refer to patients with mental health problems. This article’s analysis included these responses and did not include answers regarding other groups of patients. Other questions were directly related to patients with mental health conditions. For example, we asked the staff whether these patients are stigmatized in the ED, and if so, what the stigma is. This question allowed some participants to distinguish between their own view and their colleagues’ view, whereas other participants felt more comfortable sharing their own perspective and beliefs when responding to the broader question regarding the department’s personnel. For the latter, notes such as “it’s not stigma, it’s reality” were common. The interview guide is enclosed as Supplement 2. The interviews took place in 2019 (9 January – 15 May), were conducted in Hebrew, lasted 20–94 minutes, and were recorded, transcribed verbatim, and anonymized. The recordings and transcripts are stored securely on the researchers’ computers.

### Data analysis

The texts were analyzed using thematic analysis [53,54]. This included an iterative search procedure for expressions and ideas. Interviewees’ responses were coded and analyzed inductively according to key themes that emerged from the data, without applying a pre-determined analytic framework. We identified significant and recurrent themes in the data that represent common perceptions and experiences among the participants.

The thematic analysis included several stages. We began with open coding, in which we broke the data into discrete segments and assigned codes to identify emerging themes. This process involved labeling and categorizing the data without relying on predetermined frameworks, allowing insights to develop inductively. The open coding was conducted independently by two analysts, and the results were then compared. In most cases, the coding was similar, and we resolved differences by mutual agreement.

Subsequently, we conducted axial coding, which involved identifying connections between the initial categories, refining their definitions, and combining overlapping or related categories to form more coherent themes. This process helped us explore relationships among categories in terms of context, conditions, actions, and consequences. We then chose core categories that comprised the meaningful thematic clusters around which we organized the findings [53,54].

Throughout this process, we utilized a code book, which included definitions and examples of the codes, and we kept decision trails to ensure the accuracy and consistency of the codes. Peer debriefing, involving scrutiny by additional researchers, was also applied. Thematic saturation was assessed using Guest et al.’s method [55]. Finally, after we formed the conceptual descriptions and explanations, we reread the raw data to confirm that the analysis did not depart from the data.

The qualitative interviews with the staff members are part of a broader mixed methods study in this ED that also included a quantitative survey and qualitative interviews with patients [56,57]. While this article’s findings focus on the staff, the data gathered on the patients served as useful background information. Additionally, the broader study examined issues pertaining to ethnic and religious groups of ED patients, namely Arab and Jewish ultra-Orthodox patients [58], and addressing the Israeli-Palestinian conflict [59].

### Authors’ reflexivity

Our research team is characterized by diverse perspectives in several ways. First, our team is multidisciplinary and comprises medical anthropologists (ZO and MDF), an emergency medicine physician (EAA), and a psychiatric hospital nurse (LJ). This allowed us to understand the field and the studied phenomena from varying academic and clinical viewpoints. Second, the team includes Israeli and American members, with vast experience in studying and working in EDs in both countries, conducting qualitative, quantitative, and mixed-methods studies. This provided a broad comparative perspective. Third, while three of us were external to the examined ED, one (EAA) worked as a physician in this ED at the time of the interviews (and did not conduct interviews) and worked as a physician in another ED in Jerusalem during the analysis and writing phases. He had a “lived familiarity” with and a priori knowledge of the participants’ emic discourse, while the medical anthropologists retained an outsider’s critical, etic perspective [60]. The psychiatric nurse, who works in a different hospital in Jerusalem, may be considered “an outsider within” [61]. Taken together, these diverse positionalities, backgrounds, and qualifications have been useful. We could be familiar with the field and have access to data, but also maintain a critical point of view and ask sensitive questions [60,62,63].

### Trustworthiness

The trustworthiness of this research was ensured by meeting the following four criteria: credibility, transferability, dependability, and confirmability [64–66]. To achieve credibility, we relied on an accepted research method that has been successfully employed in previous studies in EDs, i.e., semi-structured interviews with staff. We applied investigator triangulation by involving several researchers in the analysis. Credibility was further strengthened by EAA’s sustained engagement with this ED. It was also supported by our ongoing practice of reflexive self-analysis, including the maintenance of reflexive fieldnotes.

Transferability was reinforced by offering comprehensive background information on the phenomenon under investigation to establish the study’s context. This facilitates potential application to other contexts and allows for comparative analysis by future researchers. In addition, providing richly detailed methodological information, including the data collection methods, enables other researchers to compare or replicate our study [64–66].

Dependability was ensured through the concurrent and independent open coding conducted by two team members, as well as peer debriefing, involving scrutiny by researchers who were not involved in the interviews. Additionally, reflexive auditing and a detailed methodological account were implemented. Finally, confirmability was achieved by grounding the findings exclusively in the data collected from this study, thereby minimizing potential researcher bias. Keeping decision trails also contributed to confirmability, as it allowed us to verify that the findings were not shaped by researcher bias but were instead grounded in the data [64–66].

## Results

Our analysis revealed that ED staff in this Jerusalem hospital faced significant challenges treating patients with mental health conditions. The analysis generated four main themes: navigating stigmatizing views of people with mental illness, inadequate professional skills for treating and managing mental health, organizational and structural constraints, and proposed solutions (Table 1). The data suggest that treatment of mental health in the ED could be improved by training, increased support from psychiatric personnel, and better linkages to community care.

**Table 1:** Themes and subthemes of the findings.

	Themes	Subthemes
1	Navigating stigma	The characterization and recognition of stigma
		The consequences of stigma
2	Staff’s professional skills	Insufficient capacity and low confidence
		Difficulties in communication
		The implications of insufficient skill
3	Organizational and structural constraints	Limited psychiatric services
		The structure of ED care and time constraints
		Frequent ED users and social determinants of health
4	Solutions and resources	Adapting spaces for mental health patients
		Increasing empathy and understanding
		Limiting the scope of ED treatment for mental health

### Navigating stigma

#### The characterization and recognition of stigma.

Interview participants identified multiple manifestations of stigma toward people with mental illness in the ED, including a tendency to diminish the seriousness of mental health emergencies and view individuals with mental illness as irrational, dangerous, and their treatment an inappropriate use of ED resources. While participants acknowledged being influenced by these stigmas, many sought to recognize the suffering of patients with mental illness and to provide appropriate care.

Describing existing stigmas, Student 2 said, “Many times there’s some kind of generalization, [the assumption that] a person who has […] a behavioral disorder then must also have a low level of intelligence, which many times is not the case.” Certain behaviors associated with mental illness including aggressive speech and actions were seen as dangerous, and staff responded by avoiding these patients and labeling them as difficult. Nurse 9 said, “I don’t relate that well to this field, to patients with mental illness. Especially those who are aggressive and violent, because I cannot foresee their reactions and I cannot communicate with them. […] So there is a stigma, which in my opinion has to do with patients who are loud, violent, or cannot control their reactions.”

Participants reported that mental health emergencies and symptoms were often regarded as not genuine medical issues and as inappropriate for the ED. Nurse 1 stated, “There are those who have mental illnesses – bipolar disorder and all kinds of disabilities – and it’s difficult to get along with them because they make it more difficult for us. They do whatever they please, walk around, they are independent and they come back and forth, come back every other day, so actually they are more difficult to deal with. They don’t really need treatment – they come because they need a place where they will receive attention.” Some participants attributed mental health symptoms to problematic personalities, rather than illness. Student 3 discussed how she struggles with this perception: “It is simply difficult to tell myself that it’s the illness and not the person; it’s sort of different from a physical ailment.”

A small number of participants felt that ED staff could avoid stigmatizing patients by adhering to the professional standards of healthcare. Nurse 2 said, “There is a stigma among the population in general. I think that among those in the healthcare professions, there is no stigma.”

#### The consequences of stigma.

Staff members reflected on the potential adverse medical implications of the stigma they described. Many participants noted that due to the disregard for the seriousness of mental health emergencies, patients are deprioritized during triage and face long wait times. Student 1 connected stigma to treatment delay: “I think there’s something very crucial about the stigma attributed to such people. […] It’s as though staff refuse to approach and treat them first; instead, they just let them wait at triage.” Nurse 6 acknowledged feeling inclined not to treat mental health patients, while also recognizing their need for care: “For me, it’s hard because, on the one hand, I say I don’t have the energy to provide care. On the other hand, they are miserable and they need to be here in the hospital.”

Participants recognized that discounting and deprioritizing patients with mental illness create a risk of overlooking a serious and acute physical illness, which the patient may not have been able to communicate due to their mental health condition. For mental health patients who make frequent ED visits, Nurse 8 explained, “It’s like the story of the boy who cried ‘Wolf!’, but one day they will show up with something real. They are often pushed aside and then someone discovers that they are truly sick this time, but that’s after they showed up a hundred times before without any reason […]. We no longer insert an IV cannula or take a blood sample from these patients.” Similarly, Physician 2 stated, “One day, they will show up with some kind of condition and you might not notice.” Nurse 5 acknowledged that when psychotic patients present with disordered speech, ED staff, “Take what they say with a grain of salt – there is a tendency not to take them as seriously as we should.” Despite this tendency, she advocated equal treatment: “A good caregiver will have the same exact attitude towards everyone.”

### Staff’s professional skills

#### Insufficient capacity and low confidence.

Interviews revealed a perceived lack of adequate preparation and clinical skills among ED staff in treating and managing patients with mental illness. For example, staff described difficulties in recognizing and diagnosing specific psychiatric conditions. This lack of capacity caused some staff to feel overwhelmed when caring for mental health patients. Participants also expressed low confidence in their ability to manage psychiatric cases. Physician 3 stated, “There is difficulty understanding […] the patient’s basic condition, why he arrived, getting the medical history, communicating with the patient, getting to understand what his problem is, and explaining to him what you’re going to do. […] As a healthcare provider, I do not feel comfortable with these patients because their behavior or what is normal is different in their case.” This physician demonstrated the sense of uncertainty and discomfort staff feel when lacking the clinical skills needed to interpret psychiatric presentations.

Participants explained that this inadequacy stems from a lack of focused training on mental health within emergency medicine. Physician 8 pointed out that training in emergency medicine underemphasizes mental health, stating that trainees may be “very good at taking care of people who are very sick, and they won’t have training in taking care of people with mental illness.” This suggests that even well-trained emergency staff may feel unprepared for mental health cases, due to systemic gaps in medical education and specialization.

#### Difficulties in communication.

A significant subtheme in the interviews was that ED staff perceived patients with mental illness as poor communicators and therefore difficult to diagnose and treat. Some symptomatic patients are withdrawn and uncommunicative while others are hyperverbal and express disordered speech. Nurse 8 said, “They are not able or don’t know how to express themselves and I don’t have the time and I know that they show up for no reason. It always stresses me out, dealing with these people.” Relatedly, Physician 2 stated, “In general, these patients express themselves less clearly and then it’s difficult to know exactly where the problem is and to focus on it.” Student 2 described his view that the level of skill for communicating with mental health patients varied across ED staff: “You need skills to be able to communicate with these people; there are those who have the skill and they manage. Those who do not cannot manage. Some staff members even have emotional difficulties – the moment they come into some kind of conflict or contradiction, they are no longer able to proceed.”

The lack of skill for communicating with mental health patients reinforces a stigmatizing perception of these patients as untrustworthy and uncooperative. Nurse 7 described how a lack of ability to decipher mental health complaints leads to distrust: “Their complaints are less focused, so we tend to distrust them.” One Employee stated, “They don’t always hear or understand what you’re saying; they aren’t always ready to cooperate because they are in their own loop.”

#### The implications of insufficient skill.

Participants demonstrated a clear understanding of the negative consequences and risks associated with inadequate clinical capacity in mental health. One major concern they raised was that difficulties in communicating with and diagnosing mental health patients in the ED can increase the risk of overlooking acute medical conditions. Physician 1 stated, “The patients with whom one cannot communicate or receive precise and reliable information constitute the most difficult cases, in which you have to use all of your coping methods to understand what exactly is happening and whether this patient is actually in physical distress, that is, a physical condition that requires immediate treatment or a mental issue that also apparently requires immediate treatment.”

The lack of skill and confidence in managing mental health patients also leads to emotional strain among the ED staff. Nurse 5 said, “They drive you up the wall. […] Sometimes they drive you nuts and you have no more patience and it affects your tolerance, and with all the workload we have, you just can’t stand it. You explain things once, twice, three times; that’s enough.” Relatedly, Nurse 6 said, “Sometimes, they really get on your nerves. You know they will take up a lot of your time and they have needs and they make so many demands. We have lots of patients to treat and sometimes it’s also an emotional burden, facing so many demands – it’s simply difficult.”

Finally, the inadequate capacity to address mental health led to the perception that the ED was an inappropriate setting for such patients to receive care. This view was summarized by Student 1: “The hospital is not a place where they can be treated; the place to treat them is a place with psychiatric services. […] I will attend to a medical need but I cannot address this patient’s psychiatric problems. […] I don’t know how to cope with that; I don’t have the tools to deal with psychiatric disabilities. And truly, there are loads and loads of cases of psychiatric patients in the emergency department who don’t get what they came for.”

### Organizational and structural constraints

Interview participants identified a range of organizational, structural, and social constraints limiting their ability to effectively treat mental health in the ED, including a lack of psychiatric specialists and limited connection to psychiatric services. Participants also reported that the fast-paced and chaotic atmosphere of the ED was stressful for patients and reduced the capacity for rapport-building. They also referred to the impact of social determinants of health, such as homelessness and social isolation.

#### Limited psychiatric services.

The hospital where we conducted research does not have a psychiatry department but has a Psychiatry Consultation Unit that collaborates with the ED. Moreover, this hospital does not have a residency program in psychiatry and consequently does not have in-house 24/7 psychiatrists. After 4 PM, the attending psychiatrist is available only on-call (i.e., not on-site), which may result in a longer wait before the patient’s first contact with a psychiatrist. Along with the inadequate staff training, the absence of on-site psychiatrists after 4 PM contributed to a lack of readily available psychiatric expertise.

Furthermore, the lack of a psychiatry department means that patients cannot be admitted to this hospital for mental illness. Physician assistant 2 said, “There is no psychiatric ward here; we have a few psychiatrists who come to consult. […] If you need to admit someone, you need to send them to psychiatric hospitals.” Nurse 6 described how the lack of a psychiatry department meant that ED staff could face a dead end when determining the next steps for care: “Someone with an internal problem is admitted to the internal medicine ward, and someone with an orthopedic problem is admitted to the orthopedic department. But what do you do with a psychiatric patient with mental illness? Where should they be sent? We don’t have the proper place to admit them and that’s very difficult.”

In addition to the lack of psychiatric expertise, Nurse 1 reported that the psychiatric patients’ “permanent medications are not available in the ED; they are not part of our standard supply. So often we end up destabilizing them a bit when they show up at the ED. That doesn’t seem to help.”

Together, these barriers create a situation where, as Student 3 described, “I just don’t think that the emergency department is capable of treating them. […] If someone comes during a psychotic episode that suddenly erupted, there is nothing we can do.”

#### The structure of ED care and time constraints.

The structure of the ED, designed for the stabilization of acute physical illness, was seen as detrimental to care for patients with mental illness. Several participants reported that time constraints associated with providing emergency care were not compatible with the needs of patients with mental illness, who often need extended periods of engagement and rapport-building. Student 2 said, “The emergency department does not really address the needs of these people because there simply isn’t time. […] We face a shortage of resources; often we can’t even find a quiet separate space for them. The ED is a difficult place even for those who are healthy.” The time constraints and fast-paced care processes created difficulty in building rapport, as noted by Nurse 1: “We do not have a trusting relationship with them and we cannot establish any interaction.” Nurse 7 also reported that the structure of ED care can be difficult for patients with mental illness: “Treating them is very difficult, especially in the ED. […] We have fewer resources, a smaller team, the beds are less comfortable. That is, everything is less than what makes sense for treating this type of patient, who often needs constant attention.”

#### Frequent ED users and social determinants of health.

Social factors outside of the hospital such as homelessness and social isolation worsened patients’ mental health conditions and impacted care in the ED. Stigma was heightened for patients who were homeless or used substances, as providers felt more negatively towards these patients and were more likely to disregard their complaints. This perception was applied particularly to patients who visited the ED frequently. As Nurse 6 reported, “They return for no good reason. They are homeless with mental disorders who come back without any cause.” Such patients may have challenges accessing regular care in the community and therefore seek care in the ED. While these patients may need care, ED staff often saw them as a nuisance and overlooked the structural causes of their visits. This view was reflected in Nurse 5’s statement: “They don’t really need treatment and it takes away treatment time from other patients. […] Alcoholics, homeless people, people who want to come to the hospital for their dialysis – I think they simply lie down in the street and are picked up by an ambulance and that’s how they get a ride to the hospital.”

Social isolation also impacts care processes for people with mental illness. For example, Nurse 5 explained how “Sometimes they arrive alone. […] If I want to discharge someone, I’m not sure where he came from, whether he lives in an assisted living facility or not, or if he cannot go home because he cannot take care of himself. You know, you always have to involve a social worker.”

### Solutions and resources

#### Adapting spaces for mental health patients.

Some interview participants offered specific solutions for improved ED care. One suggestion was to offer specialized, calmer spaces in the ED for mental health patients: “Place them in a quieter area, if possible, secluded with a curtain” (Nurse 5). Another suggestion was to create alternative destinations for mental health patients who would benefit from support and observation but do not need medical treatment in the ED: A place “where they could come and eat and we could sit and talk with them, give them a place to rest, where volunteers would keep them company. […] They need a framework that is similar to hospitalization, where they can stay many hours and be observed for a long period; where people are constantly passing by and they can see other people and make personal connections with those around them” (Nurse 9). This suggestion reflects the concept of a stabilization center which has been proposed by US federal agencies as a key component of the continuum of psychiatric care [67].

#### Increasing empathy and understanding.

Staff members identified empathy, understanding, warmth, and patience as essential strategies for improving ED care for patients with mental health conditions. One Employee explained: “These patients need a little bit of warmth, a little bit of patience. […] It seems to me that they mainly need people to understand them – their place and needs, where they come from. Sometimes we don’t see the full picture and only notice the person who is angry or yelling. They need to be understood, actually.” This and other participants emphasized the importance of understanding the broader context, including the patient’s complex life trajectories and social history, as a key to a successful treatment.

#### Limiting the scope of ED treatment for mental health.

Another perspective proposed restricting the circumstances under which patients with mental illness are treated in the general ED, instead directing them to alternative treatment settings. For example, Physician 5 explained: “In my opinion, as soon as someone comes and says that he or she wants either to commit suicide or to kill someone, that’s it – my part is done.” According to this participant, in these cases, the patient should be treated exclusively “in a psychiatric department or a psychiatric hospital” where “the patient is observed and cared for.”

## Discussion

This qualitative article aimed to illuminate the perceptions and experiences of ED clinicians providing care to patients with mental health conditions in Israel. The findings point to substantial challenges faced by the staff, including stigmatizing attitudes, insufficient training, and various organizational, structural, and resource constraints. Overall, our findings confirm and extend prior research on ED clinicians’ perspectives on providing mental health care.

First, we found that providers navigated a high level of stigma towards people with mental illness. Other studies have found that, for ED staff, negative feelings often arise even before meeting the patient, based on a psychiatric diagnosis only [68]. Further, due to the staff’s fear and stigma, and the perception that people with mental illness may be dangerous, many patients are subject to social control measures based on anticipated behavior [9]. Due to the stigma towards people with mental illness, staff viewed patients as manipulative, seeing their complaints as disingenuous, inappropriate, or self-imposed [9,21]. These views may also exacerbate patients’ internalized stigma [69]. Our study demonstrated that many providers were aware that negative perceptions of patients seeking mental health care were potentially stigmatizing and harmful, and in some cases tried to mitigate the effects of stigma while also acknowledging the continued influence of stigma. Our study participants recognized that stigmatizing views of patients could lead to diagnostic overshadowing, which in this context means the misattribution of a patient’s physical symptoms to a mental disorder, which may result in inadequate or delayed treatment [28,68]. Staff also realized that these stigmas could lead to premature closure, i.e., failing to consider other possibilities after an initial diagnosis is made [68]. This awareness among staff is extremely important, as it can motivate them to take action to improve the existing situation.

Similar to prior studies, ours also found that staff frequently viewed patients with mental illness as inappropriate users of the ED [9]. They also assumed limited responsibility for patients with mental health problems, who were even viewed as a distraction from the staff’s perceived main purpose, which is providing care for patients with acute physical illnesses [26]. Hence, a perceptual transformation is needed so that staff will fully acknowledge their professional and ethical responsibility for patients presenting with mental health conditions. Staff should also gain greater awareness of the rights of these patients, as articulated in the United Nations Convention on the Rights of Persons with Disabilities and within the local legal, social, and clinical contexts [70–73].

One of the reasons for this common perception is the staff’s lack of proficiency and experience in mental health. Our findings show that many ED staff did not have the knowledge and skill required to provide adequate care for patients with mental health issues. Studies have demonstrated positive results from providing additional mental health training for staff. Existing interventions in EDs typically take the form of daylong educational workshops. Usually, there is an immediate improvement in confidence in assessment [21]. ED providers often saw education on patient-provider relationships as an effective intervention to reduce stigma and increase provider empathy [74], preferring in-person, in-hospital teaching [25]. EDs in Israel should consider implementing daylong workshops for all staff, as well as longer training for selected staff. Moreover, curricula for nursing qualification and specialization in emergency medicine should incorporate dedicated instruction on the care of patients with mental health conditions. This topic should also be emphasized in both emergency medicine specialty training and board examinations. Another effective strategy is community-engaged education for medical and nursing students, such as volunteering with individuals with mental health conditions [63,75].

Additionally, given that the ED serves as the first point of care for many people experiencing mental health issues, the ED could enhance referral pathways to community services [8]. For example, protocols for care plans that follow patients into the community, and standardized procedures for communication from community to hospital may reduce repeat ED visits and improve the quality of care [76]. These steps demonstrate the need to adopt a broader systemic approach in which ED care is part of a long-term, well-coordinated, more effective mental health treatment.

This research also reasserts the need to consider the recommendations of recent studies for a close collaboration between the emergency and psychiatric departments, more support of mental health specialists in the ED, working in interprofessional care teams, as well as organizational changes in the ED, such as providing calm environments, having a mental health liaison nurse, and introducing standardized action protocols [5,7,12,22,25,27–29,68,76]. Other proposed organizational quality measures include audits of mental health care, reviews of serious cases, monitoring of patient feedback [9], and advanced tools and decision support systems to reduce diagnostic errors due to overshadowing [68]. Furthermore, reflective practice groups led by mental health professionals can provide support and guidance for ED staff [26]. Staff can also work alongside experts by experience, incorporate interventions by certified peer recovery support specialists, and be exposed to patient self-advocacy groups to reduce stigma [9,77].

Furthermore, there is a striking need to address the root causes of the problem on the systemic level by implementing large-scale policy changes and investments in expanding and improving mental health community services [34], providing patients with alternative sources of care, such as “safe spaces” and community crisis centers, including “crisis cafes” [5,68,78]. Psychiatric decision units, which are short-stay alternatives to psychiatric inpatient services for people experiencing mental health crisis, can positively impact ED mental health presentations [79,80].

Another crucial systemic change is increasing staff-to-patient ratios in EDs [68]. This solution is more expensive than the others and therefore may be less feasible. However, hiring mental health technicians (also known as behavioral health technicians) is less expensive than hiring physicians and nurses. This position can be further developed and integrated into Israeli EDs. Systemic changes to care delivery in the ED, the hospital, and the community will lead to lasting improvements in the health outcomes of people seeking mental health care in the ED. Staff and patients should be consulted on the changes so that the care meets their needs and aligns with what they value.

Finally, there is a need for new clinical guidelines and interventions to effectively respond to new mental health challenges, such as the growing number of emergency department presentations for suicide-related concerns among youth [5,81]. A recent systematic review found that EDs are unable to meet the mental health needs of young people attending EDs for self-harm and suicidal ideation [5]. In England, for example, different stakeholders – people who attended the ED for self-harm, carers, ED practitioners, and liaison psychiatry practitioners – agreed that the system is failing people who self-harm. These people are often excluded from services and can only access crisis support, resulting in inefficient cycles of attending the ED. EDs prioritize formulaic risk assessments, which patients perceive as not addressing their needs [6].

Hence, EDs should conduct more person-centered, therapeutic risk assessments, building a human connection, validating patients’ distress, and acknowledging suicidality and self-harm [6,82]. This will generate shared understanding, make patients more hopeful, and encourage help-seeking [82]. Additionally, there is a need for proper resourcing for implementing clinical guidelines on treating people presenting to EDs with self-harm and suicidal ideation [83]. Staff training is required to change stigmatizing attitudes towards self-harm [6]. In Israel, these recommendations are particularly significant due to the expected increase in self-harm and suicidality because of the war.

### Limitations

This study has several limitations. It focused on a single ED located in Jerusalem, which has unique characteristics, e.g., a significantly lower representation of Arab staff compared to the national average. This may limit the generalizability of the findings to other hospitals in Israel. Qualitative studies in additional EDs in Israel and other countries are needed. Moreover, quantitative research based on surveys and medical records may complement the results of the present research. Further, qualitative interviews and focus groups with patients with mental health conditions who visited Israeli EDs will provide a broader perspective on the issues at stake.

The study sample included participants from a range of professions, each with different medical backgrounds and varying levels of experience working with mental health patients. Since resilience and professional experiences may differ according to role and years of service, this diversity represents a potential source of variation. However, our analysis did not distinguish between professional roles or levels of experience.

Additionally, in qualitative research, translating participants’ statements may lead to the loss of cultural nuances and non-verbal expressions. This can affect the interpretation of findings and may limit the depth of the results. Finally, the interviews were conducted before the current war. It is worthwhile to conduct interviews during and after the war to identify changes in ED practices and staff experiences and perceptions.

## Conclusion

Israel has faced a decades-long shortage of mental health care providers, a problem that was exacerbated following the COVID-19 pandemic. The current crisis in mental health services, further intensified by the war, has far-reaching implications for EDs, complicating the challenges of ED staff in caring for patients with mental health conditions. The findings of this study help understand these challenges. These include stigmatizing and potentially detrimental perceptions of patients with mental illness, who are often seen as a burden rather than a part of the ED’s responsibility. Many ED staff feel unskilled to provide effective care to these patients. However, staff members are reflexive, cognizant of the problems, and willing to partake in various interventions to provide better care to patients with mental health conditions. These interventions would be crucial in the post-war era.

Our findings point to several avenues for improving ED care for patients with mental illness. These include training staff to recognize stigma towards patients with mental illness and to recognize how social factors drive repeated ED visits. Clinical training for staff could help them recognize specific conditions and provide strategies for communicating with symptomatic patients and for de-escalating mental health crises. Improved confidence and skill in managing patients with mental illness could decrease the emotional strain associated with the frustration reported by participants. Our findings also suggest that ED care could be improved by strengthening collaboration and referral pathways to psychiatry and community-based behavioral health. Systemic changes beyond the ED are crucial to mitigating the expected post-war crisis in Israeli EDs.

## Supporting information

S1 File Inclusivity-in-global-research-questionnaire. (DOCX)
